# Epistaxis, or Bleeding of the Nose

**Published:** 1887-07

**Authors:** B. L. Cetlinski


### EPISTAXIS, OR BLEEDING OF THE NOSE.

By B. L. Cetlinski, M. D.

A very interesting communication has been made lately to the Medical Academy of Paris, by Professor Verneuil, concerning this malady. There are two kinds of epistaxis, one, acute or spontaneous, the other sympathetic or indicating the existence of a more severe derangement of the system. The first is to be met with in perfectly healthy persons of a sanguine temperament. The state of puberty is more especially subject to this disease, but it is more frequently met with in boys than in girls. At this age any slight cause determines the bleeding of the nose, as, for instance, exposure to ardent rays of the sun, exercise, fatiguing walks, intellectual labor, and sometimes even a simple strong emotion is able to induce epistaxis ; then again, the influence of various seasons of the year, a sudden shock, alcoholic drinks, or exciting articles of food ; a hurt or a scratch in the interior of the nose, causing a rupture of some capillary in the lining membrane, which causes the nasal hemorrhage. This kind of epistaxis appears, for the most part, spontaneously, but sometimes it is preceded by headache or other slight symptoms of a cerebral congestion, which disappears gradually and leaves no bad consequences. [Hence the popular belief that bleeding of the nose is healthy, and should not be interfered with.]—Translator.

Epistaxis is sometimes hereditary, and it is frequently observed that in such cases the nasal hemorrhage, which may appear severe during infancy, disappears entirely in a few years. It is sometimes difficult to distinguish the acute epistaxis from the sympathetic, when the principal disease is still in a latent state. The sympathetic epistaxis appears at the beginning of typhus fever, or accompanies some eruptive fevers, jaundice or yellow fever, scorbutic affections, heart, liver, lung and kidney complaints.

In the greater number of such cases the bleeding stops by itself, in consequence of the blood becoming coagulated, which does the service of a tampon or plug, in which case the bleeding reappears when the bloody clot is displaced by sneezing or cleansing. When the bleeding is slight, it ceases by itself, but when it is excessive and stopped with difficulty, it may be followed by serious consequences.

Nobody could stand a loss of blood of twenty to thirty kilograms without injury. Such epistaxis results mostly in real anaemia, great paleness, cold extremities, cold sweat and syncope, ending in death if not attended to in time.

I have stated that in many cases it is necessary to stop the hemorrhage as soon as possible, but, as we will show, this is not always easy to accomplish. When the hemorrhage ceases of itself it is usually the case that the flow diminishes gradually, the heat in the extremities reappears the pulse rises and the exhaustion of the patient is much overcome. This kind of epistaxis, active and spontaneous, should not be resisted, especially in patients who are strong, robust and plethoric.

In patients who are full blooded and threatened with cerebral congestion, it is sometimes better to allow the bleeding to follow its free course for a brief period, and be ready to stop it at the proper time. It often happens that the patient dies of anaemia in consequence of too much confidence in nature's effort to stop the flow. When the epistaxis is due to constitutional causes, there being are no organic lesions, and the flow not abundant but frequent, we must try to prevent the return of the attack by proper hygiene, frugal living and the employment of bitters and other tonics.

When it is caused by heart disease or liver and kidney affections, then it should be treated with digitalis, ergot or sulphate of quinine, rather than by local treatment.

In the absence of a physician, it would be well to place the patient in a cold room, the head elevated, the arms and legs bent upward and tied, and the body placed, in that position, in hot water for a few minutes, keeping the head covered with cold compresses, and to induce the patient to practice breathing freely.—*Doctor Z., from Courrier Des Etats-Unis.*

---

The simplest way to stop the hemorrhage is to plug the nostrils with soft linen well saturated with "Ponds's Extract" or hamamilis virgin.—*The Translator.*

